# Incidence, Pattern, and Possible Risk Factors for Nasopharyngeal Cancer in the Qassim Region

**DOI:** 10.7759/cureus.49355

**Published:** 2023-11-24

**Authors:** Lulwah S Alhumaidan, Yasmeen A Alfouzan, Thana M Alsenaid, Khadijah I Alburayt, Ruba M Altowayan, Seba M Alraddadi, Waleed Alhazmi

**Affiliations:** 1 College of Medicine, Unaizah College of Medicine and Medical Sciences, Qassim University, Qassim, SAU; 2 College of Medicine, Qassim University, Qassim, SAU; 3 Otolaryngology, Head and Neck Surgery, College of Medicine, Qassim University, Buraidah, SAU

**Keywords:** ent, ذancer incidence, qassim, saudi arabia, nasopharyngeal cancer

## Abstract

Objective

To evaluate and assess the incidence, patterns, and possible risk factors for nasopharyngeal cancer among the Qassim population.

Methods

A retrospective study was conducted at Dr. Suliman Al-Habib Hospital in Qassim. The study included all previously confirmed cases of nasopharyngeal neoplasms between 2016 and 2022. Data was collected retrospectively from the hospital’s health record, including the patient's demographics, clinical presentation, and comorbidities. IBM Corp. Released 2017. IBM SPSS Statistics for Windows, Version 25.0. Armonk, NY: IBM Corp. was used for data entry, management, and analysis.

Results

A total of 84 records of patients were included in the study. Males (n=55, 65.5%) were more than females (n=29, 34.5%). The majority of the patients (n=51, 62%) were aged 31-60 years. About 32 (38.0%) patients were obese. Most of the patients (n=49, 58%) presented with malignant neoplasms of the nasopharynx. The most frequent clinical presentations were nasal obstruction, runny nose, hearing loss, and neck mass.

Conclusion

The incidence rate of nasopharyngeal cancer was significantly higher in males compared to females (p=.003). There was no statistically significant association between age and comorbidity and the development of nasopharyngeal cancer.

## Introduction

Nasopharyngeal carcinoma (NPC) is a cancer that originates from the nasopharyngeal epithelium. Nasopharyngeal carcinoma is uncommon when compared to other cancer types. The incidence rates of NPC deaths have significantly dropped, which may be attributable to a combination of changes in lifestyle, population screening, and treatment advancements [1]. NPC is regarded as an uncommon cancer in many countries around the world, although it is substantially more common in particular populations, such as those in Southeast Asia, the Middle East, and North Africa, with a peak in Southern China [2]. This distinctive racial/ethnic and geographic distribution of NPC suggests that both genetic and environmental factors are important contributors to its genesis. Regardless of the frequency or geographical spread, the emergence of nasopharyngeal carcinoma (NPC) has been linked to the interplay of several environmental and genetic elements. These factors encompass Epstein-Barr virus (EBV) chromosomal abnormalities, inappropriate hypermethylation of promoters, and other genetically related concerns. Nasopharyngeal carcinoma (NPC) exhibits a notably elevated survival rate of roughly 95% when detected at stages I or II. Conversely, the prognosis for patients diagnosed with NPC at stages III or IV is considerably poorer, with survival rates just exceeding 50% [3].

The prevalence of this cancer in particular geographical areas raises the possibility that genetic and/or stable environmental risk factors play a significant role in the disease's onset. The etiology of NPC is further complicated by the Epstein-Barr virus's (EBV) contribution to pathogenesis [4]. In most populations, the incidence of NPC among men is double or triple that of NPC among women [5]. The risk of NPC is inversely correlated with age at first regular cigarette smoking and positively correlated with average daily cigarette consumption [6,7]. Chinese-style salted fish eating is listed by the IARC as a known cause of NPC [8]. Inverse relationships between the incidence of NPC and dietary consumption of carrots and other red inflammatory and/or anti-nitrosation actions, fruits, and other red, orange, and yellow vegetables point to potential antioxidants and anti-vegetarians [9-12]. Respiratory tract disorders discovered connections between NPC risk and favorable outcomes [13-16].

Notably, HNCs compose 6% of all malignancies identified annually in Saudi Arabia, while the NPC represents 33% of all head and neck cancers that are diagnosed [3]. The prevalence of nasopharyngeal cancer in the Saudi population fluctuates from year to year; however, there was a sharp rise in cases between 2017 and 2016. The fact that there is a correlation between age and incidence for both men and women show that, in Saudi Arabia, nasopharyngeal cancer primarily affects the elderly [17]. According to a recent study, the age-standardized rate (ASR) of nasopharyngeal cancer in Saudi Arabia varies by region. The study, which used data from Saudi Cancer Registry reports for the period of 2007 to 2016, discovered that there is a significant regional variation in the incidence of nasopharyngeal cancer. The Qassim region had the highest ASR of male cases of nasopharyngeal cancer, followed by the Riyadh region. Jazan and the northern regions, in contrast, had the lowest ASR. The Jouf region had the largest ASR of females with nasopharyngeal cancer, followed by the Qassim region [17].

Since 1990, Saudi Arabia's yearly death rate per 10,000 NPC patients has decreased by 65.9%, an average of 2.8% annually. In addition, the mortality rate of NPC has changed over time for men and women in particular age groups. In Saudi Arabia, the death rate for men starts to increase at age 80 or older, whereas the death rate for women reaches its peak at this age [3].

## Materials and methods

A retrospective observational study with a quantitative approach was employed. The study involved all patients with nasopharyngeal neoplasm who were diagnosed at Al-Habib Hospital in Qassim, Buraydah, between 2016 and 2022. Data were collected from the electronic medical records. All cases of nasopharyngeal carcinoma following otorhinolaryngology clinics or other clinics were included. The criteria for inclusion are all subjects with complete medical record data, including these variables: age, gender, nationality, presenting symptoms, the primary location of the neoplasm, type of neoplasm, grade, and risk factors. Exclusion criteria are medical records that have missing or incomplete data for the variables needed in this study. 

The data have been collected via clinical diagnosis as the primary method. All of the patients' data were reviewed and then entered into tables. Collecting patient data in our study was carried out by reviewing the medical records using a structured data collection sheet containing patients' sociodemographic data, presenting symptoms, current diagnosis with type of neoplasm, and their risk factors. Possible risk factors were patient comorbidities such as asthma, diabetes, hypertension, and smoking. According to the 2005 Tumor Classification of the World Health Organization, the histological findings of nasopharyngeal carcinoma (NPC) are classified. This includes three subtypes: keratinizing squamous cell carcinoma, nonkeratinizing squamous cell carcinoma, and poorly differentiated carcinoma.

Data entry and statistical analysis were done IBM Corp. Released 2017. IBM SPSS Statistics for Windows, Version 25.0. Armonk, NY: IBM Corp. We calculated the frequencies and percentages of all studied variables, like the demographic characteristics of patients. To identify the significant risk factors, a cross-tabulation by chi-square test, ANOVA test, and Pearson correlation were conducted to analyze the F-value, p-value, and the relationship among variables. A p-value less than 0.05 was considered statistically significant.

Ethical approval was obtained from the Institutional Review Board committee at Dr. Suliman Al Habib Medical Group before conducting the study, and all data was kept confidential and will be used only for research purposes. 

## Results

A total of 84 records of patients were included in the study. Males (n=55, 65.5%) were more than females (n=29, 34.5%). The majority of the patients (n=51, 62%) were aged 31-60 years, with a mean age of 43.5 years. Most patients had a BMI of 30 and above (n=32, 38.0%). The vast majority of the patients attended the ENT clinic, constituting 74 (88.0%), while the rest of the patients attended general surgery clinics, plastic surgery clinics, spinal surgery clinics, neurosurgery clinics, and pediatric clinics. The majority of the patients (81, 96.4%) were Saudi nationals, while only three (3.6%) had unspecified nationalities. Table 1 summarizes the social characteristics of the patients.

**Table 1:** Social characteristics of the participants (N=84) Social characteristics of patients are presented in frequencies (n) and proportions (%) BMI: Body Mass Index

Variables	Category	Frequency and Proportion n (%)
Gender	Male	n=55 (65.5%)
	Female	n=29 (34.5%)
Age	0-30 years	n=13 (16%)
	31-60 years	n=51 (62%)
	61-83 years	n=18 (22%)
BMI	Less than 18.5	n=4 (4.8%)
	18.5-24.9	n=25 (29.8%)
	25.0-29.9	n=23 (27.4%)
	30.0 and above	n=32 (38.0%)
Clinic	ENT Clinic	n=74 (88.0%)
	General Surgery Clinic	n=2 (2.4%)
	Spinal Surgery Clinic	n=1 (1.2%)
	Neuro Surgery Clinic	n=1 (1.2%)
	Plastic Surgery Clinic	n=2 (2.4%)
	Pediatric Clinic	n=1 (1.2%)
	Pulmonary Clinic	n=2 (2.4%)
	Internal Medicine Clinic	n=1 (1.2%)
Nationality	Saudi	n=81 (96.4%)
	Unspecified	n=3 (3.6%)

Table 2 depicts the clinical presentation, initial presenting complaint, and histopathologic type of nasopharyngeal cancer among the patients. The majority of the patients (n=44, 52.3%) presented with malignant neoplasms of the nasopharynx, and 35 (41.7%) presented benign neoplasms of the nasopharynx. In terms of initial presenting complaints, 28 (33.3%) of the patients had nasal obstruction and a runny nose; 20 (23.8%) had hearing loss. In terms of histopathologic type, the majority of the patients (30.7%) had poorly invasive differentiated type. 

**Table 2:** Clinical presentation and histopathologic type (N=84) Clinical presentation and histopathologic type are presented in frequencies (n) and proportion (%)

Description	Frequency and Proportion n (%)
Benign neoplasm of nasopharynx	n=33 (40%)
Malignant neoplasm of nasopharynx	n=37 (45%)
Malignant neoplasm, unspecified	n=11 (13%)
Nasopharyngeal myiasis	n=1 (1.2%)
Initial presenting complains	Frequency and Proportion n (%)
Nasal obstruction	n=28 (33.3%)
Nasopharyngeal swelling	n=9 (10.7%)
Nasal discharge	n=7 (8.3%)
Hearing loss	n=20 (23.8%)
Itchiness, cough, long-term snoring	n=5 (6.0%)
Neck mass	n=11 (13.1%)
Tinnitus, ear pain	n=4 (4.8%)
Ear blockage	n=7 (8.3%)
Histopathologic type	Frequency and Proportion n (%)
Nonkeratinizing squamous cell carcinoma	n=13 (15.5%)
Moderately differentiated invasive non-keratinizing carcinoma	n=22 (26.2%)
Poorly differentiated malignant neoplasm	n=47 (56%)
Mixed keratinizing and non-keratinizing carcinoma	n=2 (2.4%)

Table 3 shows the comorbidities with nasopharyngeal cancer among the patients. The results found that more than half of the patients (n=50, 60%) who were diagnosed at Al-Habib hospital had no comorbidity, 32 (38%) were obese, seven (8.3%) had hypothyroidism, seven (8.3%) were smokers, and three (3.6%) had asthma.

**Table 3:** Comorbidities (N=84) Comorbidity with nasopharyngeal cancer is presented in frequencies (n) and proportions (%)

	Type	Frequency and Proportion n (%)
Comorbidity	Asthma	n=3 (3.6%)
	Hyperlipidemia, IHD	n=5 (6.0%)
	Obesity	n=32 (38%)
	Hypothyroidism	n=7 (8.3%)
	Smoking	n=7 (8.3%)
	Pre-diabetes	n=3 (3.6%)
	None	n=50 (60%)

Table 4 demonstrates the relationship between gender, age, comorbidity, and the development of nasopharyngeal cancer. The results established a statistically significant association between gender and the development of nasopharyngeal cancer (p=.003). There was no statistically significant association between other variables (age and comorbidity) and the development of nasopharyngeal cancer.

**Table 4:** The association between gender, age comorbidity and development of nasopharyngeal cancer Association between demographic variables and the development of nasopharyngeal cancer

		Development of nasopharyngeal cancer		
Variables	Category	High	Low	p-value
Gender	Male	47 (56.4%)	37 (43.6%)	0.003
	Female	36 (43.2%)	48 (56.8%)	
Age	0-10 years	36 (43.4%)	48 (56.6%)	0.623
	11-20 years	36 (42.7%)	48 (57.3%)	
	21-30 years	37 (43.6%)	47 (56.4%)	
	31-40 years	37 (43.5%)	47 (56.5%)	
	41-50 years	35 (42.1%)	49 (57.9%)	
	51-60 years	36 (42.5%)	48 (57.5%)	
	61-70 years	36 (42.7%)	48 (57.3%)	
	71 years old and more	35 (41.8%)	49(58.2%)	
Comorbidity	Asthma	35 (41.4%)	49 (58.6%)	0.871
	X Smoker	37 (43.6%)	47 (56.4%)	
	Hyperlipidemia, ischemic heart disease	37 (43.5%)	47 (56.5%)	
	Obesity	35 (42.1%)	49 (57.9%)	
	Hypothyroidism	37 (44.0%)	47 (56.0%)	
	Smoking	36 (42.7%)	48 (57.3%)	
	Pre-diabetes	38 (44.9%)	46 (55.1%)	
	None	47 (56.2%)	37 (43.8%)	

## Discussion

The study aims to assess the incidence, pattern, and possible risk factors for nasopharyngeal neoplasms among patients diagnosed at Dr. Suliman Al-Habib Hospital, Qassim, Saudi Arabia. The study results revealed that more than half of the patients (52.3%, n = 44) presented malignant neoplasms of the nasopharynx, and a substantial number of them (41.7%, n = 35) presented benign neoplasms of the nasopharynx. The study noted that the vast majority of the patients attended ENT clinics, constituting 88.0% (n = 74), while others attended general surgery clinics, plastic surgery clinics, spinal surgery clinics, neurosurgery clinics, and pediatric clinics. The study noted that the most common initial symptoms among the patients were bilateral nasal obstruction, runny nose, bilateral hearing loss, facial headache, and pain in the eyes, constituting 33.3% (n = 28); additionally, neck mass, tinnitus, ear pain, cough, sore throat, epistaxis, ear blockage, dizziness, as well as heartburn and nasal discharge, were common initial symptoms among the patients diagnosed at the facility. The findings were consistent with those of Suárez et al. (2010), who found nasal obstruction, hearing loss, and neck mass to be common clinical presentations related to nasopharyngeal carcinoma [18]. The findings were also supported by Jang-Chun et al. (2014), who found that patients with nasopharyngeal cancer presented with initial symptoms that included hearing loss, bilateral nasal obstruction, and neck mass [19]. The study revealed that the poorly invasive differentiated type was the most common histopathologic type of nasopharyngeal carcinoma among patients diagnosed at the facility. The study noted that more than half of the patients who were diagnosed at Al-Habib Hospital had no comorbidity, while others had various comorbidities, including hypothyroidism, infertility, smoking, hyperlipidemia, obesity, asthma, pre-diabetes, and hyperlipidemia. All the patients diagnosed at the hospital had no family history of nasopharyngeal cancer. The study conducted by Chua et al. (2016) found that the risk of developing nasopharyngeal cancer seems related to several factors, including environmental aspects, smoking, obesity, and genetic predisposition [1]. The findings of the study substantially corroborate the current findings on a number of cancer risk factors. However, the current study found no family history among the cancer patients diagnosed at Al-Habib Hospital.

The incidence rate of nasopharyngeal cancer was higher in males compared to females, with males constituting 65.5% (n = 55) and females 34.5% (n = 29). The study findings are consistent with those of the study conducted by Alsafadi et al. (2020) in Saudi Arabia, which found the incidence rate of nasopharyngeal cancer patients to be higher in males than females, with an almost 3:1 male-to-women ratio [20]. Similar results from the International Agency for Research on Cancer at WHO (WHO-IARC) that were seen in nasopharyngeal cancer patients in Kuwait found male patients to be significantly higher than females [21]. The study established a statistically significant association between gender and the development of nasopharyngeal cancer (p =.003). There was no statistically significant association between other variables (age and comorbidity) and the development of nasopharyngeal cancer. This implies that there was no significant direct impact of comorbidity or age on the development of nasopharyngeal cancer.

The major limitation of this investigation was that a significant number of the patients were excluded due to incomplete clinical data, thereby making interpretations with respect to the development of nasopharyngeal cancer limited. Also, a larger sample size would have generated more precise results, but it wouldn’t have been possible to get larger datasets considering that it was a retrospective study conducted over the past 10 years. Additionally, the study findings cannot be generalized to the entire Saudi Arabian population, given that they were conducted in only one region.

## Conclusions

Overall, the study revealed that the majority of the patients presented with malignant neoplasms of the nasopharynx. The most common initial symptoms among the patients were nasal obstruction, runny nose, hearing loss, neck mass, facial headache, and pain in the eyes. The most common histopathologic type of nasopharyngeal carcinoma among the patients was the poorly differentiated type. The study established a statistically significant association between gender and the development of nasopharyngeal cancer, with a male predominance. There was no statistically significant association between age, comorbidity, and the development of nasopharyngeal cancer. This implies that there was no significant direct impact of comorbidity and age on the development of nasopharyngeal cancer.

## References

[1] Chua MLK, Wee JTS, Hui EP, Chan ATC (2016). Nasopharyngeal carcinoma.. Lancet.

[2] Irekeola AA, Shueb RH, E A R EN (2021). Prevalence of nasopharyngeal carcinoma in patients with dermatomyositis: A systematic review and meta-analysis. Cancers (Basel).

[3] Abdullah DA, Hussain GA, Abdelbaset ME (20191). Nasopharyngeal cancer in Saudi Arabia: Epidemiology and possible risk factors. Journal of Oncological Sciences.

[4] Ye W, Chang ET, Liu Z (2017). Development of a population-based cancer case-control study in southern china. Oncotarget.

[5] Bray F, Colombet M, Mery L (2021). Cancer Incidence in Five Continents Volume XI. https://publications.iarc.fr/597.

[6] Yuan JM, Wang XL, Xiang YB, Gao YT, Ross RK, Yu MC. Non-dietary risk factors for nasopharyngeal carcinoma in Shanghai, China. https://onlinelibrary.wiley.com/doi/10.1002/.

[7] Cheng YJ, Hildesheim A, Hsu MM (1999). Cigarette smoking, alcohol consumption and risk of nasopharyngeal carcinoma in Taiwan. Cancer Causes Control.

[8] FR L (2012). TOBACCO SMOKING - personal habits and indoor combustions.

[9] Warwick R A, Imrey P P, Sann Munn L, Jocelyn M A, Mimi C Y, Sham S (199877). Nasopharyngeal carcinoma in Malaysian Chinese: salted fish and other dietary exposures. J Cancer.

[10] Ning JP, Yu MC, Wang QS, Henderson BE (1990). Consumption of salted fish and other risk factors for nasopharyngeal carcinoma (NPC) in Tianjin, a low-risk region for NPC in the People's Republic of China. J Natl Cancer Inst.

[11] Liu YT, Dai JJ, Xu CH (2012). Greater intake of fruit and vegetables is associated with lower risk of nasopharyngeal carcinoma in Chinese adults: a case-control study. Cancer Causes Control.

[12] Polesel J, Serraino D, Negri E (2013). Consumption of fruit, vegetables, and other food groups and the risk of nasopharyngeal carcinoma. Cancer Causes Control.

[13] M C Y, C C M, W X C, F S Y, B E H (1988). Preserved foods and nasopharyngeal carcinoma: a case-control study in Guangxi, China - PubMed. Cancer Res.

[14] Yu MC, Garabrant DH, Huang TB, Henderson BE (1990). Occupational and other non-dietary risk factors for nasopharyngeal carcinoma in Guangzhou, China. Int J Cancer.

[15] Chung SD, Wu CS, Lin HC, Hung SH (2014). Association between allergic rhinitis and nasopharyngeal carcinoma: A population-based study. Laryngoscope.

[16] Kim HJ, Ahn HS, Kang T, Bachert C, Song WJ (2019). Nasal polyps and future risk of head and neck cancer: A nationwide population-based cohort study. J Allergy Clin Immunol.

[17] Kabli AF, Miyajan KF, Alqurashi AS (2022). Trends in the Incidence of Nasopharyngeal Cancer in Saudi Arabia Across One Decade (2007 to 2016). Cureus.

[18] Suárez C, Rodrigo JP, Rinaldo A, Langendijk JA, Shaha AR, Ferlito A (2010). Current treatment options for recurrent nasopharyngeal cancer. Eur Arch Otorhinolaryngol.

[19] JA-JA L, JI-JI H, YE-YE J (2014). Comparisons of quality of life for patients with nasopharyngeal carcinoma after treatment with different RT technologies. Acta Otorhinolaryngol Ital.

[20] Alsafadi N, Alqarni MS, Attar M, Mgarry R, Bokhari A (2020). Nasopharyngeal cancer: prevalence, outcome, and impact on health-related quality of life at Princess Norah Oncology Center, Jeddah, Saudi Arabia. Cureus.

[21] Whelan SL, Parkin DM, Masuyer E (1990). Pattern of cancer in five continents.

